# Medical and Physical Intelligence

**Published:** 1822-01

**Authors:** 


### Medical and Physical Intelligence.

### 1. *Flatted Sculls.*

ONE of the most curious problems in craniology is that which relates to the configuration impressed upon the bones of the head by art.

It has been long understood that the human head was capable, by compression, of assuming almost any form, between a trencher and a sugar-loaf.

The sculls received by Dr. MITCHILL, from Colonel T. H. PERKINS, are of a remarkable character, and were brought from the Columbia river, on the N.W. coast of America. They were compressed as if by the action of a heavy weight, or strong mechanical power, acting downward and backward from the frontal bone. This pressure must have been begun in early infancy, and continued for the whole time, during which the bones were yielding and flexible. The consequence is, that the os frontis is depressed almost to an angle of eight or ten degrees. The parietal bones are crushed down in a similar manner, and brought as nearly as possible to a level surface. In two of the sculls, the coronal and sagittal sutures are much less distinct than common, and, in some parts, almost obliterated.

The most evident effect of this violence to the cranium is the protrusion of the parietal bones behind the ears, to make room for the squeezed and displaced brain. This is such as to impart to the head a deformed, monstrous, or rickety appearance.

It is understood that the mechanical power, thus applied to the head, widens or enlarges its base, as well as its summit. The individuals who undergo the operation, are the children of the more considerable and important families. The lower and plebeian orders are not honoured with the distinction of *flat heads*. Such a scull, of the rounded form which nature gave it, accompanies the others, and makes a striking contrast. Living witnesses attest, that the men, whose heads have been severely pressed and tortured, possess a full proportion of mental and corporeal vigour.

The case is very curious in a physiological and moral, as well as in an anatomical, point of view.—*New-York Repository, April* 1821.

### 2. *Remedy for Psora.*

DR. EDMUND PORTER, of Hunterdon Co. N. J., states that he has obtained great success in the treatment of psora, by the internal exhibition of the spirits of turpentine, even after all the ordinary remedies for the disease had failed. He used it in doses of from five to thirty drops daily, until the disease entirely disappeared, which was usually in the course of fourteen days.—*New-York Rep.*

### 3. *Prophylactic against Hydrophobia.*

CANTHARIDES, dissolved in vinegar, are usually employed, in Hungary, as a remedy against the bite of mad dogs, in the case of both man and animals. This preparation is taken internally.—*Allgem. Mediz. Zeitung, April* 1821.

### 4. *Longevity.*

THERE is a person of the name of Brunner, living now in Altenstein in Bavaria, who is 115 years of age. He was born on the 16th of April, 1706, at Trappstadt in Franconia. His principal occupation has been that of a day-labourer; but he has now been a pauper some time. His food is very plain. He was married, for the third time, in his 97th year, to his present wife, who lives with him. He is in the habit of taking a walk daily for several hours; and appears, in every respect, to be in perfect health.—*Allgem. Mediz. Zeitung.*

### 5. *Nasal Polypi.*

COUNT PIETRO MOSCATI, of Milan, has invented an ingenious instrument for applying a ligature to polypi of the nose that hang pendulous in the throat, which is entirely free from every inconvenience belonging to the instruments now in use for that operation.—*Giornale di Fisica & di Chimica.*

### 6. *Cholera Morbus at Samarang.*

ABOUT a month ago, a very malignant disorder manifested itself among the native population at Samarang. The Javanese, Malays, and Chinese, who were attacked with it, died, for the most part, after twelve hours' indisposition; many fell down dead suddenly after a very short indisposition. This extraordinary mortality excited the attention of the faculty, and convinced them that the cholera morbus had reached the island. It soon attacked the Christian population; and, especially, was very destructive among the military. The terror and confusion became general, and were soon followed by the flight of the Europeans to the mountains and cooler districts. On some days, when the thermometer rose to 95 or 100 degrees, from 160 to 180 persons died at Samarang, as well islanders as Europeans and their descendants.

The general distribution of good medicines, and the cooler weather, have already checked this disorder. However, of a company of European hussars of eighty men, twenty-five have been buried; and, of five superior officers at Samarang, two majors, English and Depré.

For persons who have not enervated themselves by the use of strong liquors,—for those who have a good constitution and a cheerful disposition,—this disorder is not more formidable than an ordinary fever, provided they daily use the proper remedies. The poor Javanese, who wanted the inclination or the ability, have suffered the most.

The sickness broke out here a few days after it appeared at Samarang: it is, however, of a less malignant description. Of sixty Europeans, who were attacked, 16 have died. We have reason to hope that the cool weather, and the approaching rice-harvest, will wholly put an end to the sickness. Many persons chiefly ascribe it to the bad food which the Javanese of the lower classes have been obliged to use for some months.—*Private letter from Souraucasta, in Java, May* 26, 1821.

### 7. *Jennerian Festival in Berlin.*

The tenth anniversary dinner of the Jennerian Society of Berlin, for the propagation of Vaccination, was celebrated, a few months back, with much splendour, under the presidency of Dr. Hufeland. An account was laid before the meeting of the number of children vaccinated during the year 1819 throughout the Prussian States, amounting to 337,344, without including Dusseldorf, Juliers, Magdeburg, Berlin, and Aix-la-Chapelle, from which places returns had not then been received.—*Allgem. Mediz. Zeit.*

### 8. *Royal mark of favour to a physician.*

Louis, &c.—On the report of our Minister Secretary of State for the Home Department.

Wishing to recompense the zeal which Dr. Bally has manifested at Barcelona, in devoting himself to the study and treatment of the contagion which spreads its ravages there, we have ordered and do order as follows :—

Art. 1. The Sieur Bally, Doctor of Medicine and Member of the Royal Academy of Medicine, is appointed a Knight of the Royal Order of the Legion of Honour.

2. Our Minister Secretary of State for the Home Department, and the Grand Chancellor of the Legion of Honour, are charged with the execution of the present Ordinance.

(Signed) LOUIS.
(Countersigned) SIMEON.

*Given at the Tuileries, Dec. 5.*

### 9. *Ornithology.*

Mr. R. Robinson, of Derby, has a beautiful living specimen of that scarce bird the great-eared owl, which is not much inferior in size to the eagle. Its head is very large, and is adorned with two erect tufts, more than two inches long, which stand just above each eye; its bill is strong and much hooked, its eyes large and of a bright yellow colour; the whole plumage is of a rusty brown, finely variegated with black and yellow lines; its legs are remarkably strong, and covered to the claws with a thick close down. It is a young bird, and was taken in Russia.—*New M. Mag.*

### 10 *Electro-Magnetism.*

In our department of Collectanea we have given a short abstract history of this new science. In continuation of it, we are happy to relate, some new experiments. M. le Chevalier Yelin, a learned Bavarian, discovered, some time ago, that needles of steel become magnetic when placed in a glass tube surrounded with a metallic spiral, and when electrical sparks, or the charge of a Leyden battery, were transmitted along the spiral. When the spiral was turned from left to right, and the sparks taken from the positive conductor, the end of the needle which points to it becomes a south pole, and the other the north pole, and *vice versa.* If a third needle, reckoning from its middle, is surrounded in a spiral manner with waxed taffetas, then the poles appear at the points where the spiral begins and ends. If, instead of a spiral wire, a rod of metal is extended along the glass tube, the steel needle, placed within, becomes feebly magnetic after several strong electrical discharges. The poles of a magnetic needle were entirely reversed by several electrical discharges along the spiral. —*New M. Mag.*

### 11. *Liverpool Ophthalmic Infirmary.*

In our last number we inserted a report of the cases treated at the Institution for curing diseases of the eye at Liverpool, under the head of "Liverpool Eye Infirmary." This might lead some of our readers to suppose that there is in that town but one institution of the kind; whereas, the fact is, that an Infirmary for the treatment of diseases of the eye does exist in Liverpool, as well as the institution before named, but under the management of different medical officers, with whose names we have been favoured by one of the surgeons, together with a list of the cases treated at that establishment between the 1st of July, 1820, and the 30th of June, 1821, being not less than 1145; as extracted from the annual Report, which, with great pleasure, we subjoin.

*Physician*—T. S. Traill, M. D.
*Surgeons*—Mr. Christian, Mr. Jas. Dawson, Mr. T. F. Hay.

MEDICAL REPORT.

*Diseases of the Eye.*

| | | | |
|---|---|---|---|
| Ophthalmia | 239 | Ophthalmia a causis chemicis | 9 |
| ——— Erysipelatosa | 111 | Iritis | 21 |
| ——— Purulenta | 63 | Staphyloma | 11 |
| ——— Pustulosa et ulcerosa | 165 | Leucoma | 91 |
| | | Glaucoma | 3 |
| ——— Porriginosa | 56 | Hydrophthalmia | 1 |
| ——— Rheumatica | 5 | Cataracta | 22 |
| ——— Syphilitica | 6 | Asthenia Visus | 17 |
| ——— Traumatica | 9 | Amarousis | 9 |
| ——— a causis mechanicis | 24 | Anomali | 34 |

*Diseases of the Eye-lids.*

| | | | |
|---|---|---|---|
| Psorophthalmia | 60 | Conjunctiva Palpebralis Granulosa | 17 |
| Tarsi Ulcerosi | 103 | | |
| Ptosis | 1 | Abscessus, Tumores, Verrucæ, &c. | 19 |
| Ectropion | 10 | | |
| Entropion | 3 | | |

*Diseases of the Lachrymal Passages.*

Inflammatio, Abscessus, Distensis, Obstructio ........ 36

Total 1145

### METEOROLOGICAL JOURNAL.

**By Messrs. William Harris and Co. 50, Holborn, London.**

***From November* 20 *to December* 19, *inclusive.***

| Day of Month. | Moon. | Rain gauge | THERM. | | | BAROM. | | DeLuc's Hygro. | | WIND. | | ATMOSPHERIC VARIATION. | | |
|---|---|---|---|---|---|---|---|---|---|---|---|---|---|---|
| Nov | | | | | | | | | | | | | | |
| 20 | | | 50 | 52 | 52 | 29.97 | 29.78 | 54 | 54 | WSW | WSW | Fine | Cloud. | |
| 21 | | | 50 | 48 | 42 | 29.65 | 29.88 | 58 | 56 | NW | NW | Rain | Fine | Cloud |
| 22 | | | 46 | 54 | 46 | 29.55 | 29.61 | 57 | 56 | SW | W | Rain | Fine | |
| 23 | | .20 | 52 | 49 | 39 | 29.71 | 29.95 | 56 | 57 | W | NNW | Cloudy | Rain | Fine |
| 24 | | .25 | 46 | 50 | 43 | 29.72 | 29.74 | 57 | 56 | WSW | WNW | Rain | Cloud. | Fine |
| 25 | ● | .10 | 45 | 55 | 45 | 29.63 | 29.57 | 56 | 60 | NW | SW | Fine | | Rain |
| 26 | | | 53 | 55 | 49 | 29.38 | 29.26 | 62 | 60 | WSW | WSW | Cloudy | Rain | Cloud. |
| 27 | | | 48 | 50 | 36 | 29.51 | 29.90 | 56 | 55 | WNW | W | Cloudy | Sho'ry | Fine |
| 28 | | .27 | 43 | 50 | 49 | 29.81 | 29.74 | 55 | 60 | WSW | WSW | Cloudy | Rain | Fine |
| 29 | | | 52 | 52 | 48 | 29.69 | 29.61 | 65 | 60 | SW va. | SW va. | Cloudy | | Fine |
| 30 | | .09 | 50 | 52 | 49 | 29.55 | 29.47 | 55 | 57 | W var. | W | Cloudy | | Rain |
| Dec | | | | | | | | | | | | | | |
| 1 | | .20 | 52 | 53 | 41 | 29.63 | 29.81 | 53 | 55 | NW va. | W | Fine | Rain | |
| 2 | ☾ | | 43 | 49 | 39 | 29.90 | 30.02 | 54 | 53 | W var. | W | Cloudy | Fine | |
| 3 | | .16 | 41 | 48 | 36 | 29.88 | 29.80 | 55 | 55 | W var. | WNW | Cloudy | Rain | Fine |
| 4 | | .26 | 40 | 48 | 37 | 29.82 | 29.76 | 56 | 60 | SW | W | Fine | | Rain |
| 5 | | .41 | 41 | 48 | 36 | 29.87 | 29.95 | 55 | 55 | W | NW | Showery | R.&Li. | Fine |
| 6 | | | 39 | 42 | 40 | 30.20 | 30.30 | 53 | 55 | NE | SE | Fine | | |
| 7 | | .10 | 40 | 49 | 44 | 30.19 | 30.11 | 57 | 55 | S | SSW | Showery | | Cloud. |
| 8 | | | 43 | 53 | 49 | 30.04 | 30.12 | 62 | 63 | W | W | Cloudy | | |
| 9 | ○ | | 50 | 51 | 41 | 30.10 | 30.07 | 65 | 65 | SW | SW | Showery | | |
| 10 | | .08 | 42 | 46 | 38 | 30.00 | 29.95 | 61 | 61 | W | W | Fine | | Sho'ry |
| 11 | | | 39 | 48 | 40 | 30.21 | 30.35 | 60 | 56 | S | SSE | Cloudy | Fine | Cloud. |
| 12 | | | 43 | 52 | 41 | 30.20 | 30.09 | 61 | 63 | S | S | Cloudy | Fine | Cloud. |
| 13 | | .06 | 42 | 51 | 43 | 30.00 | 30.02 | 62 | 63 | SE | SE | Cloudy | | Sho'ry |
| 14 | | .16 | 43 | 50 | 46 | 30.03 | 29.97 | 61 | 61 | S | SSW | Cloudy | Rain | Cloud. |
| 15 | | | 47 | 51 | 46 | 29.99 | 29.85 | 63 | 60 | S | S | Cloudy | | |
| 16 | ☽ | | 48 | 52 | 49 | 29.71 | 29.63 | 61 | 58 | W | SW | Very fine | | Cloud. |
| 17 | | .11 | 50 | 53 | 49 | 29.52 | 29.32 | 63 | 62 | SW | SW | Rain | | Cloud. |
| 18 | | .18 | 51 | 52 | 45 | 29.25 | 29.13 | 61 | 57 | SW va. | SW | Rain | | |
| 19 | | .13 | 46 | 49 | 42 | 29.21 | 29.33 | 63 | 65 | SW | W | Rain | Sho'ry | |

The quantity of rain fallen in the month of November, was 3 inches and 59-100ths.

